# Safety outcomes of sodium-glucose cotransporter-2 inhibitors in patients with type 2 diabetes and other risk factors for cardiovascular disease: a systematic review and meta-analysis

**DOI:** 10.1097/XCE.0000000000000284

**Published:** 2023-05-08

**Authors:** Radhika Deshpande, Raj Patel, Manjari R. Regmi, Mohsin Salih, Robert Kropp, Basma Al-Bast, Muhammad A. Sheikh, Andrew Sagalov, Abhishek Kulkarni, Momin Siddique, Shruti Hegde, Mukul Bhattarai

**Affiliations:** a Department of Internal Medicine, Southern Illinois University School of Medicine; b Division of Cardiology, Southern Illinois University School of Medicine, Springfield, Illinois, USA

**Keywords:** adverse reactions, heart failure, outcomes, sodium-glucose cotransporter-2 inhibitors, type 2 diabetes

## Abstract

Sodium-glucose cotransporter-2 inhibitors (SGLT2-Is) have emerged as standard therapy for heart failure. We aim to assess the safety of SGLT2-Is in patients with a high risk of cardiovascular disease.

**Areas covered:**

An electronic database search was conducted for randomized control trials comparing SGLT2-Is to placebo in patients with a high risk of cardiac disease or heart failure. Data were pooled for outcomes using random-effect models. The odds ratio (OR) and 95% confidence interval (CI) were used to compare eight safety outcomes between the two groups. The analysis included ten studies with 71 553 participants, among whom 39 053 received SGLT2-Is; 28 809 were male and 15 655 were female (mean age, 65.2 years). The mean follow-up period was 2.3 years with the range being 0.8–4.2 years. The SGLT2-Is group had a significant reduction in AKI (OR = 0.8;95% CI 0.74–0.90) and serious adverse effects (OR = 0.9; 95% CI 0.83–0.96) as compared to placebo. No difference was found in fracture (OR = 1.1; 95% CI 0.91–1.24), amputation (OR = 1.1; 95% CI 1.00–1.29), hypoglycemia (OR 0.98;95% CI 0.83–1.15), and UTI (OR = 1.1; 95% CI 1.00–1.22). In contrast, DKA (OR = 2.4; 95% CI 1.65–3.60) and volume depletion (OR = 1.2; 95% CI 1.07–1.41) were higher in SGLT2-Is group.

**Expert opinion/commentary:**

The benefits of SLGT2-Is outweigh the risk of adverse events. They may reduce the risk of AKI but are associated with an increased risk of DKA and volume depletion. Further studies are warranted to monitor a wider range of safety outcomes of SGLT2-Is.

## Introduction

The disease burden of heart failure (HF) is approximately 1–2% worldwide, and the clinical course of HF leaves patients with a poor quality of life. In addition, HF remains a common cause of hospitalization and mortality worldwide [1]. There have been several large clinical trials in the recent literature evaluating sodium-glucose cotransporter-2 (SGLT2-Is) inhibitors as a potential therapy for HF with reduced (and more recently preserved) ejection fraction (HFrEF, HFpEF). One recent study revealed that the use of SGLT2-Is was associated with a decreased risk for cardiovascular death or hypertensive HF by 33%, with a number needed to treat 5.7 (*P* < 0.001) [2].

SGLT2-Is act on the sodium-glucose cotransporter-2 channel, a near kidney exclusive channel, and are one of the newer therapies with renoprotective and cardioprotective profiles [3]. They also confer a favorable impact on many diabetic endpoints, such as slowing the progression of albuminuria, decreasing HbA_1_c, and increasing weight loss [4–6].

In 2019, the US Food and Drug Administration (FDA) approved SGLT2Is as a therapy to reduce the risk of hospitalization for HFrEF [7]. After the EMPEROR-Preserved trial, SGLT2-Is have also become essential therapy for HFpEF [8–10]. Given the large amount of data published in recent years, there is a need to pool all data from randomized control trials (RCTs) to generate the most up-to-date safety profile for these drugs, since the adverse effects of SGLT2-Is are not clearly understood. This is especially important given SGLT2-Is are emerging as standard therapy.

The true significance and magnitude of adverse effects of SGLT2-Is such as volume depletion, infections, amputations, fractures, and diabetic ketoacidosis (DKA) are not elaborately studied previously. Due to the lack of this data, there may be ambivalence with the initiation of this protective and impactful class of medication for fear of causing harm. We aim to comprehensively analyze the safety outcomes of SGLT2-Is. This study is unique in that we include ten of the latest high-quality RCTs with large sample sizes whereas prior metanalyses could not include data from more recent trials. The large sample size allows for greater generalizability and results are more reliable, especially when evaluating for safety outcomes. An additional benefit of this study is the exploration of various strategies to reduce the risk of adverse events.

## Methods

### Selection of studies

**Fig. 1:**
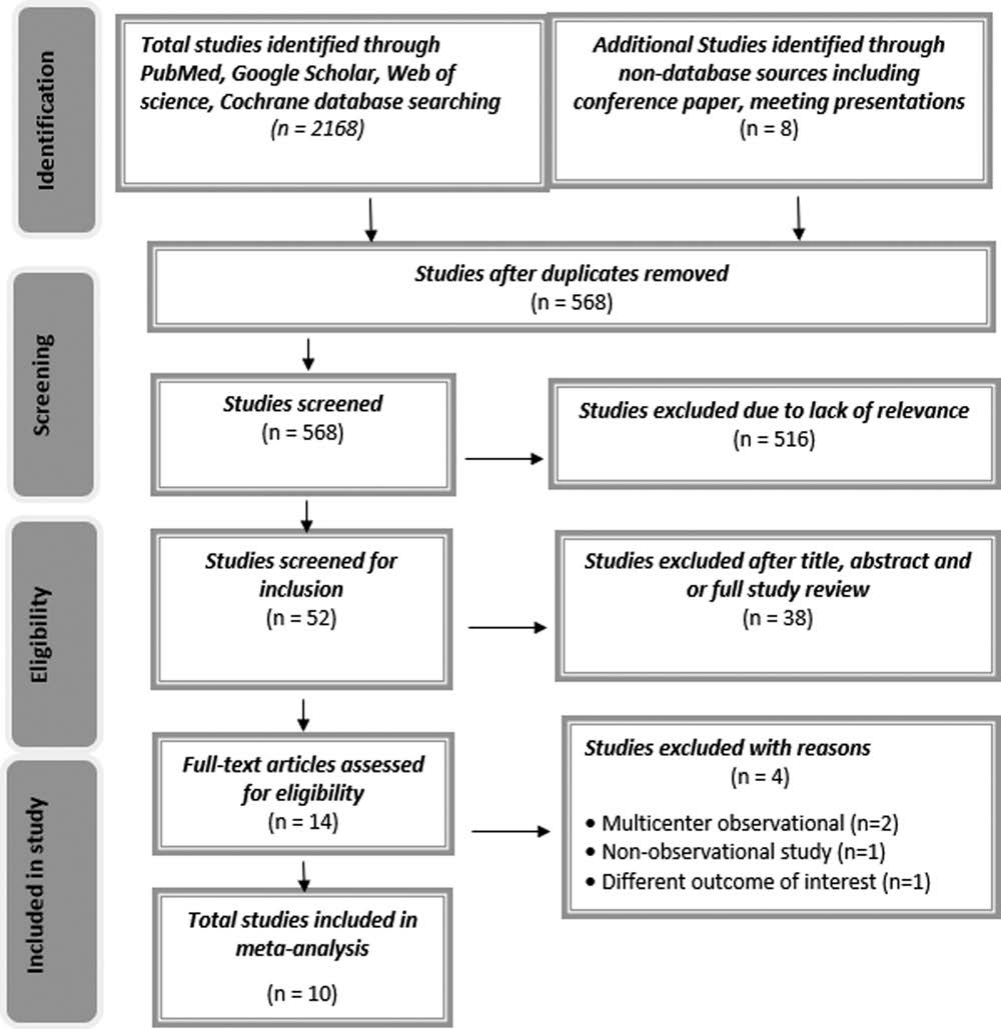
PRISMA flow diagram demonstrating study search strategy on SGLT2 Inhibitors and cardiovascular outcomes. PRISMA, preferred reporting items for systematic reviews and meta-analyses; SGLT2-I, sodium-glucose cotransporter 2 inhibitors.

A search of Google Scholar, PubMed, Web of Science, and Cochrane was conducted from database inception to 10 January 2021. The conference proceedings, ClinicalTrials.gov, and reference lists of published trials, reviews, and meta-analyses were also searched. Keyword and medical subject heading (MSH) search terms included SGLT2-Is and cardiovascular outcomes, dapagliflozin and cardiovascular outcomes, canagliflozin and cardiovascular outcomes, empagliflozin and cardiovascular outcomes, and ertugliflozin and cardiovascular outcomes. In addition, different combinations of MSH search terms were used. Final eligible studies were selected with the consensus of all authors. The preferred reporting items for systematic reviews and meta-analyses (PRISMA) reporting guidelines were used to provide the search strategy to obtain all eligible studies. The PRISMA flow diagram and the reasons for study exclusion are provided in Fig. 1.

The following standard criteria were set to select eligible RCTs comparing outcomes of SGLT2-Is use versus a placebo control group. The baseline characteristics included established atherosclerotic cardiovascular disease (ASCVD), a high-risk of ASCVD, or HF with or without kidney disease and diabetes. In addition, the trial reported cardiovascular outcomes. After removing the duplicates, a total of 568 studies were screened. Ten high-quality studies were eligible for the meta-analysis, and multiple observational studies were excluded from the meta-analysis. The study did not include several trials that have yet to release their final results. Ten RCTs were included in the study [5,6,9,11–17]. Risk-of-bias assessment in data extraction was resolved with the consensus of all authors.

### Quality assessment

The methodological quality of RCTs was assessed using a modified Jadad Score. A score of 0 to 8 quantified the quality of each trial; high-quality studies (score ≥3) were included, as shown in supplement, Supplemental digital content 1, http://links.lww.com/CAEN/A40.

### Outcomes

Both efficacy and safety were analyzed by performing a meta-analysis. Efficacy outcomes were already published [2]. The safety outcomes were DKA, fracture, amputation, volume depletion, acute kidney injury (AKI), hypoglycemia, ASAEs, and urinary tract infection (UTI). The adverse outcomes were defined in the individual original trials. The definition of AKI in the majority of trials was a doubling of creatinine from baseline. The exception to this is two trials, SOLOIST-WHF and Declare TIMI 58. SOLOIST-WHF defined AKI as >30% increase in baseline and Declare TIMI 58 defined AKI as an increase in serum creatinine above 50% from baseline. The data were abstracted by observing event rates in different groups from each trial. The exclusion and inclusion criteria of each study, including outcomes, were all presented separately.

### Data analysis

Cochrane review manager software (RevMan 5.3, The Nordic Cochrane Center) was used to perform pairwise analysis. The data from the included trials were used to calculate the odds ratio (OR) and 95% confidence interval (CI) comparing the intervention and the placebo groups. The analysis of all outcomes was performed using a Mantel-Haenszel equation and the random-effects model. A 2-sided *P* < 0.05 was considered statistically significant for all analyses. Heterogeneity was tested using I^2^ and χ^2^ tests. I^2^ index values of 25–50% were considered low heterogeneity; 51–75%, moderate heterogeneity; and greater than 75%, high heterogeneity. Publication bias was evaluated by funnel plot.

## Results

Ten studies with 71 553 participants were included, among whom 39 053 received SGLT2-Is; 28 809 were men, and 15 655 were women [mean age, 65.2 (range, 61.9–70.0) years] (Table 1). Race and ethnicity were defined in the original trials and were categorized as Asian, Black, or other (6900 participants) and White (26 646 participants) (the category ‘other’ was not specified consistently). Regarding age, 16 793 patients were younger than 65 years, and 17 087 patients were 65 years or older. The mean follow-up was 2.3 (range, 0.8–4.2) years.

**Table 1:** Salient features of study and participants of included studies

	EMPA-REG-Outcome, Zinman *et al.*	CANVAS and CANVAS-R, Neal *et al.*	CREDENCE, Perkovic *et al.*	DAPA-HF, McMurray *et al.*	DECLARE TIMI 58, Wiviott *et al.*	DAPA-CKD, Heerspink *et al.*	EMPEROR-Reduced, Packer *et al.*	VERTIS-CV, Cannon *et al.*	SOLOIST-WHF, Bhatt *et al.*	SCORED, Bhatt *et al.*
Year	2015	2017	2019	2019	2019	2020	2020	2020	2020	2020
Study type	RCT (placebo control)	RCT (placebo control)	RCT (placebo control)	RCT (placebo control)	RCT (placebo control)	RCT (placebo control)	RCT (placebo control)	RCT (placebo control)	RCT (placebo control)	RCT (placebo control)
Type of SGLT2-I	Empagliflozin	Canagliflozin	Canagliflozin	Dapagliflozin	Dapagliflozin	Dapagliflozin	Empagliflozin	Ertugliflozin	Sotagliflozin	Sotagliflozin
(dose in mg)	10 and 25	100 and 300	100	10	10	10	10	5 and 15	200–>400	200–>400
SGLT2-I (n)/placebo (n)	2333/4687	5795/4347	2202/2199	2373/2371	8582/8578	2152/2152	1863/1867	5499/2747	608/614	5292/5292
Follow-up (years)	3.1	2.4	2.62	1.52	4.2	2.4	1.33	3.5	0.75	1.33
Age (mean, SD)	63 (8.7)	63.3 (8.3)	63.0 (9.2)	66.3 (10.9)	63.9 (6.8)	61.85 (12.1)	66.85 (11)	64.4 (8.05)	70 (NA)	69 (NA)
Female (%)	28.5	35.8	33.9	23.4	37.4	33.1	23.9	29.9	33.7	44.9
Established cardiovascular disease (%)	7020 (100%)	6656 (65.6%)	2223 (50.5%)	4744 (100%)	6974 (40.6%)	1610 (37.4%)	3730 (100%)	8238 (100%)	1222 (100%)	NA
History of CHF	706 (10.1)	1461 (14.4)	652 (14.8)	4744 (100%)	1724 (10.0)	468 (10.9)	3730 (100%)	1958 (23.8)	1222 (100%)	3283 (31.0)
Mean LVEF (%)	27.5			31					35	
eGFR < 60 mL/min per 1.73m^2^	1819	2039	2592	1226	1265	3850	1799	1807	NA (Avg 49.7)	NA
HgbA1c	8.1 (0.8)	8.2 (0.9)	8.3 (1.3)	NA	8.3 (1.2)	NA	NA	8.2 (0.95)	7.1 (NA)	8.3 (NA)
BMI (SD)	30.6 (5.3)	32 (6)	31.3 (6.2)	28.2 (6)	32 (6)	29.5 (6.2)	27.9 (5.4)	31.95 (5.5)	30.8 (NA)	31.8 (NA)

CHF, congestive heart failure; LVEF, left ventricular ejection fraction; RCT, randomized control trial; SGLT2-I, sodium-glucose cotransporter-2 inhibitors.

**Fig. 2:**
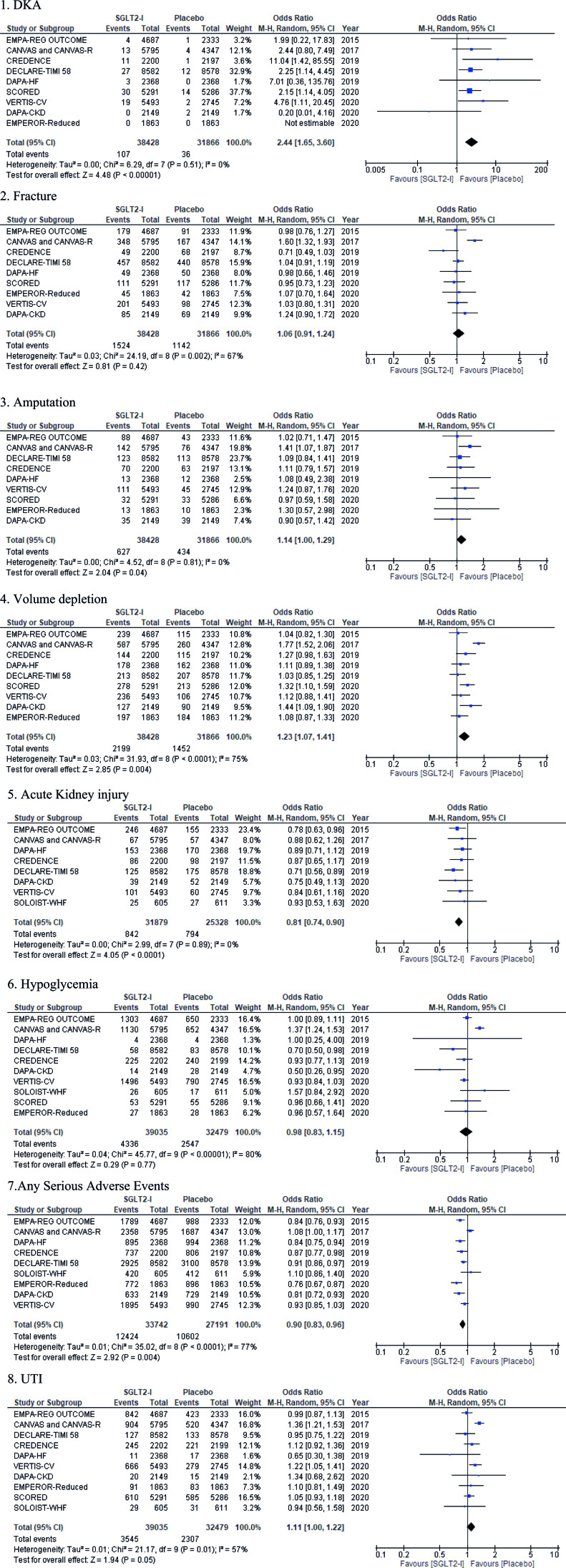
Forest plot showing common safety outcomes of SGLT2-I in patients with a high risk of cardiovascular disease. DKA, diabetic ketoacidosis; SGLT2-I, sodium-glucose cotransporter-2 inhibitors; UTI, urinary tract infection.

The frequency of AKI was statistically significantly lower in the SGLT2-Is group when compared to placebo group (2.6% vs. 3.1%, OR = 0.81; 95% CI 0.74–0.90) (Fig. 2). The incidence of serious adverse events (ASAEs) was statistically significantly lower in the SGLT2-Is group than in the placebo group (37% vs. 39%, OR = 0.9; 95% CI 0.83–0.96).

The number of DKA events was statistically significantly lower in the placebo group than in the SGLT2-Is group (OR = 2.4; 95% CI 1.65–3.60). Volume depletion was statistically significantly more frequent in the SGLT2-Is group when compared with placebo group 5.7% vs. 4.6% (OR = 1.2; 95% CI 1.07–1.41).

The incidence of hypoglycemia between SGLT2-Is (7.8%) and placebo (11.1%), demonstrated no statistically significant difference (OR = 0.98; 95% CI 0.83–1.15). There was no statistically significant difference in the degree of fracture between SGLT2-Is group (4%) compared to placebo group (3.6%) (OR = 1.1; 95% CI 0.91–1.24). Though the frequency of amputation was higher in SGLT2-Is arm (2.2% vs. 1.4%) than in placebo arm, as the 95% CI included 1.00, it was deemed statistically insignificant (OR = 1.1; 95% CI 1.00–1.29). This was the same case with UTI, which had increased events in the SGLT2-Is group as compared to placebo (9.1 vs. 7.1%). However, as the CI included 1.00, the difference was statistically non-significant (OR = 1.1; 95% CI 1.00–1.22).

## Discussion

After pooling safety outcomes of ten RCTs enrolling high-risk patients of cardiac disease, the significant findings of this study are: (1) SGLT2-Is are associated with a decreased risk for AKI and ASAEs. (2) SGLT2-Is are not associated with increased risks of fracture or hypoglycemia. (3) SGLT2-Is are associated with an increased risk of diabetic ketoacidosis and volume depletion. (4) SGLT2-Is had an increased trend toward amputation and UTI. However, it was statistically insignificant.

The renal benefits of SGLT2-Is are well known. Though there are concerns about the increased risk of AKI with SGLT2-Is, pooled results from 8 RCTs found a decrease in risk for AKI (2.64% vs. 3.13%, OR = 0.81; 95% CI 0.74–0.90). Trials EMPA-REG OUTCOME and DECLARE TIMI 58 found a decrease in risk for AKI with SGLT2-Is as compared to placebo while the remaining trials found no associated risk for AKI [6,17]. AKI and chronic kidney disease (CKD) have complex causality. On the one hand, patients with CKD are more susceptible to AKI, presumably due to decreased glomerular reserve; however, AKI can also lead to CKD progression [18]. CREDENCE, DAPA-CKD, EMPEROR-Reduced, and EMPEROR-Preserved all found SGLT2-Is maintained renal function and prevented progression of CKD by measuring baseline eGFR over time, and they found an early dip in trend of baseline eGFR followed by decreased decline of eGFR over time as compared to placebo [8,13,15,16]. This was postulated to be secondary to reducing intraglomerular pressure per CREDENCE study [16].

Another mechanism that may help prevent the progression of CKD may be the same mechanism that reduces risk for AKI, which is by lowering serum uric acid levels. Uric acid has been linked to AKI and CKD via proinflammatory effects, hypertension, and crystal-dependent mechanisms [19,20]. EMPEROR PRESERVED and EMPA-REG OUTCOME trials found decreased level of uric acid in patients in SGLT2-Is group as compared to placebo [8,17]. SGLT2-Is lower serum uric acid by increasing glucose concentration in renal tubules, causing glucose to compete with urate for reabsorption across GLUT9b transporter. Lowering serum uric acid levels may contribute to renoprotective benefits of SGLT2-Is as well as cardiovascular benefits, since elevated uric acid has been associated with hypertension and MACE [19].

Our results were congruent with other metanalysis conducted by Qui *et al*. (2021), Pelletier *et al*. (2021), Menne *et al*. (2019), and Tang *et al*. (2017) [21–24]. Interestingly, Pelletier *et al*. (2021) and Tang *et al*. (2017) found inconsistent renal outcomes with canagliflozin, increased renal adverse effects with dapagliflozin, and decreased renal adverse effects including AKI for empagliflozin [22,24].

Despite the reduction in the incidence of AKI, pooled results of the present metanalysis found that the use of SGLT2-Is is associated with a 1.23-fold increased incidence of volume depletion. Volume depletion with SGLT2-Is occurs due to osmotic diuresis by glucosuria. Increased incidence of volume depletion can be explained by the fact that studies incorporated in the current metanalysis included patients with HF with or without CKD. Patients with HF are treated with diuretics and each study had at least 40% of patients on a diuretic at baseline. Rahhal *et al*. (2021) found that patients treated with combined SGLT2-Is and loop diuretics had greater adverse effects related to volume depletion as compared to single-agent SGLT2-Is [25]. Milder *et al*. recommended that when SGLT2-Is are added to medication regimens, diuretic doses may need to be adjusted to prevent hypotension or significant volume depletion [26]. Prior studies such as Qiu *et al*. (2021) had similar findings regarding volume depletion as ours [21]. The diuretic effect of SGLT2-Is may have beneficial effects, including reduced systolic blood pressure and decreased risk for HF exacerbation. Volume depletion can also lead to hypotension which was reported in three (SOLIST WHF, SCORED, EMPEROR PRESERVED) of the included studies [8,9,11].

Another concern with the use of SGLT2-Is and resulting glucosuria is the development of UTIs. Prior data showed inconsistent results; however, our analysis demonstrated SGLT2-Is are associated with an increased risk of UTI (9.08 vs. 7.10%) compared to placebo. The difference, however, did not attain statistical significance. As it stands, patients with diabetes have an increased propensity for UTI at baseline due to a degree of immunocompromise, urinary retention secondary to neuropathy, as well as glucose in the urinary tract due to poor glycemic control. SGLT2-Is induce glucosuria; however, patients with uncontrolled diabetes may have baseline glucosuria. This may explain why the risk for UTI is equivocal [27]. Patients who generally do not have baseline glucosuria such as well-controlled diabetic or even nondiabetic patients may have an increased risk for UTI with the use of SGLT2-Is. These findings are in accordance with the findings of prior studies including Dave *et al*. (2019) and Lega *et al*. (2019) [28,29].

The US FDA released a warning in 2015 and a revision in 03/2022, regarding the increased association of SGLT2-Is and DKA [30,31]. The mechanism of action of SGLT2-Is can lead to reduced insulin-to-glucagon ratio by inhibition of SGLT2 receptor on pancreatic islet cell, direct stimulation of glucagon release, glucosuria leading to decrease in insulin demand, and stimulation of hormones such as glucagon, cortisol, growth hormone, and catecholamines. These hormone changes cause hyperglycemia and ketogenesis. Glucosuria caused by SGLT2-Is renders blood glucose levels normal or slightly elevated, therefore, causing euglycemic DKA. This phenomenon occurs more commonly in patients with type 1 diabetes rather than type 2 [32,33]. The trials CREDENCE, DECLARE TIMI 58, SCORED, and VERTIS-CV all found a statistically significant increase in risk for DKA [6,11,12,16], whereas, EMPA-REG OUTCOME, CANVAS, DAPA-HF, and DAPA-CKD found no difference in risk for DKA with SGLT2-Is or placebo [5,13,14,17]. The mechanism of action of SGLT2-Is in the pathogenesis of DKA can account for the results of the present study, which reflect an increase in risk for DKA and no difference in risk for hypoglycemia in SGLT2-Is as compared to placebo.

DKA is triggered by precipitating factors such as acute illness, surgery, excess alcohol intake, or dehydration. This can cause a stress response and induce release of cortisol, catecholamines, and glucagon, thereby altering the insulin-glucagon ratio [33–36]. It can be difficult to identify, since DKA may present with euglycemia rather than classic signs of hyperglycemia. Therefore, patients must be educated about measures for prevention such as temporarily withholding SGLT2-Is during acute illness, dehydration, or excess alcohol consumption for 3 days prior to surgery [36,37]. Another tactic for prevention for patients treated with insulin is to refrain from reducing the dose of insulin on initiation of SGLT2-Is to ensure that the insulin glucagon ratio is maintained [33]. Due to concern for hypoglycemia, it may be tempting to reduce the dose; however, this can preclude to development of DKA.

All included studies enrolled patients with type 2 diabetes rather than type 1 diabetes in whom DKA is more common; therefore, event rates may not have generated sufficient power to produce accurate outcomes. Hence, pooling results from RCTs shows SGLT2-Is use was significantly associated with a 2.44-fold increased risk of DKA compared with placebo. When comparing our results to other studies, Caparrotta *et al*. (2021) had equivocal results which could be attributed to the decreased power of this study (5 studies) as compared to ours (10 studies) [38]. Qui *et al*. (2021) conducted metanalysis with 8 RCTs and found similar results as ours [21]. Donnan *et al*. (2019) found no significant increase in risk for DKA in pooled results of 16 RCTs which they attributed to minimal event rate with insufficient sample size of included RCTs [39]. These studies emphasize DKA is a rare, albeit serious, adverse effect of SGLT2-Is.

There must be a careful balance and monitoring on initiation of SGLT2-Is to avoid both DKA and hypoglycemia outcomes as they are both grave adverse effects. SGLT2-Is had no association with hypoglycemia in the current study after measuring pooled outcomes from 10 RCTs. The antidiabetic medications most commonly associated with hypoglycemia are insulin and insulin secretagogues [40]. Their mechanisms of action may have a role to play as no other antidiabetic medications directly interact with insulin, including SGLT2-Is, and do not pose as great of a risk for hypoglycemia. Goring *et al*. (2014) completed a metanalysis comparing antidiabetic medications (namely DPP-4 inhibitors, thiazolidinediones, sulfonylureas, and SGLT2-Is) in combination with metformin and found that SGLT2-Is had an equal or decreased risk for hypoglycemia [41].

Our results found that though there was an inclination towards increased number of events in SGLT2-Is arm as compared to placebo arm for amputation and OR was skewed towards favoring placebo, it was not statistically significant as the 95% CI included 1.00. Based upon the findings of CANVAS and CANVAS-R, the FDA issued a warning for canagliflozin specifically, not SGLT2-Is as a class, regarding increased risk for fracture and amputation [30]. In the CANVAS trial, there was an increased risk for amputation with canagliflozin than placebo, with the greatest risk in patients with a prior history of amputation or PVD. Authors in CANVAS trial did not find any explanation for why there were more associated amputations with canagliflozin than with placebo. Interestingly, other trials that utilized canagliflozin (CREDENCE) did not yield the same results that CANVAS obtained [5,16]. A recent meta-analysis including 7 RCTs had similar results to our study with overall analysis finding no association between SGLT2-Is and amputation. However, subgroup analysis indicated that canagliflozin had increased association with amputation with results primary driven by CANVAS study [42]. A study that compared SGLT2-Is with DPP4i found no difference in risk for amputation between both medications [43]. Interestingly, another comparative study found a decreased risk for amputation in SGLT2-Is when compared to sulfonylureas [44]. Diabetic patients have a predilection for amputations due to neurovascular complications with an increased amputation risk in association with diuretics [45,46]. As SGLT2-Is have a diuretic effect, there may be an increased risk for amputation. This should be further analyzed in other studies before it can be declared a valid effect of diuretics.

Our metanalysis found no statistically significant difference in risk for fracture in SGLT2-Is arm as compared to placebo as demonstrated in prior meta-analysis [47]. In the CANVAS trial, there was an increased rate of all fractures with canagliflozin than with placebo, but not in CANVAS-R [5]. A review article by Compston *et al*. elucidated that patients with type 2 diabetes have an association with fractures possibly due to factors such as obesity, falls due to hypoglycemia, and visual impairment by diabetic retinopathy/cataract, sarcopenia, and multiple comorbidities associated with diabetes (including CKD) [48]. The review article also detailed the role of anti-diabetic medication with fractures and noted increased risk of fractures associated with thiazolidinediones with inconsistent results seen with metformin, sulfonylureas, incretin-based medications, as well as SGLT2-Is [48]. The contribution to fracture risk by SGLT2-Is may be secondary to increased phosphorus reabsorption by the kidneys, causing increased release of parathyroid hormone and subsequent increase of bone resorption [49].

Serious adverse events such as death, amputation, bone fracture, DKA, serious renal adverse events, complicated genitourinary tract infection, thromboembolic events, acute pancreatitis, and renal cell or bladder cancer, as an aggregate, were found to be decreased in our study in SGLT2-Is arm as compared to placebo. Many of these were described in the above sections of the discussion.

SGLT2-Is for patients with HF are associated with mortality benefits, reduction in MACE outcomes, and reduction in cardiovascular readmissions [2]. Our study finds that SGLT2-Is are associated with a decrease in serious adverse effects as compared to placebo. Additionally, there was decreased association with AKI. We found no association with fractures, amputation, UTIs, and hypoglycemia. Ultimately, the benefits of SGLT2-Is largely exceed the risks. The strength of this study is the use of 10 large, high-quality, original RCTs which has given a large pool of data, the magnitude of which has not previously been conducted. Due to this, the results in this study carry a validity not previously demonstrated. In addition, mitigation strategies to decrease side effects and further improve safety outcomes are also discussed.

With regard to future directions, long-term safety data are lacking. This is a relatively new medication and the longest follow-up period among these RCTs is 4–5 years. Ideally, there will be a follow-up study to the RCTs to determine safety 10–20 years after regular use of SGLT2-Is. Additional side effects may be identified as SGLT2-Is use becomes more widespread. Safety endpoints such as bladder cancer, pancreatitis, and Fournier’s gangrene were not examined in this study due to limited number of events. Furthermore, use of SGLT2-Is in certain populations such as patients with renal transplant or type 1 people with diabetes has limited data as these patients were in exclusion criteria in large RCTs. Certain outcomes such as fractures, amputations, and UTI will need further exploration to clearly delineate the risk. Overall, there is a need for more RCTs in the future to further stratify the safety of SGLT2-Is as it is a mainstay therapy for CHF.

## Limitations

The limitations are that as we analyzed RCTs only with the exclusion of nonrandomized and observational studies, we had limited selection bias. All the available evidence was independently analyzed to reduce the bias. There was no access to patient-level data to perform propensity/stratified analysis which could better define differences between treatment groups with respect to patient characteristics or other personalized factors. The definition of what constitutes an outcome was variable across all studies, which may affect the assessment of the outcome. The rare side effects of SGLT2-Is such as fracture, amputation, and DKA from the included studies may not represent definitive incidence because occurrence patterns may vary in a larger population than that of the trials. Amputation and UTI had 95% CIs that included 1.00 which negated the results by making it statistically insignificant, though the overall OR and event rates were higher in SGLT2-Is group. More studies are needed to clarify this risk. Certain outcomes like Fournier’s gangrene were not included in the final analysis due to limited number of events reported in overall trials.

### Conclusion

The prevalence of HF is growing with the advancements in coronary revascularization and there is considerable morbidity and mortality associated with the condition [1]. SGLT2-Is are the newest class of medications approved for the therapy of HF as they confer a mortality benefit, reduction in MACE outcomes, and reduction in cardiovascular readmissions. Our study finds that SGLT2-Is are associated with a decrease in serious adverse effects as compared to placebo. Additionally, there was a decrease in association with AKI. When weighing the risks and benefits, the benefits of SGLT2-Is largely exceed the risks.

## Acknowledgements

Special thanks to SIU Library for the language edits.

The authors confirm contribution to the article as follows: study conception and design: Mukul Bhattarai, MD, Radhika Deshpande, MD; data collection: Radhika Deshpande, MD, Mohsin Salih, MD, Mukul Bhattarai, MD, Manjari R. Regmi, MD. Author; analysis and interpretation of results: Mukul Bhattarai, MD, Radhika Deshpande, MD, Raj Patel, MD, Manjari R. Regmi, MD, Mohsin Salih, MD, Robert Kropp, MD; draft article preparation: Mukul Bhattarai, MD, Radhika Deshpande, MD, Raj Patel, MD, Manjari R. Regmi, MD, Mohsin Salih, MD, Robert Kropp, MD, Basma Al-Bast, MD, Muhammad Adil Sheikh, MD, Abhishek Kulkarni, MD, Andrew Sagalov, DO, Momin Siddique, MD, Shruti Hegde, MD. All authors reviewed the results and approved the final version of the article.

All authors take responsibility for all aspects of the reliability and freedom from bias of the data presented and their discussed interpretation.

Disclaimer: The views expressed in the submitted article are his or her own and not an official position of the institution or funder.

### Conflicts of interest

There are no conflicts of interest.

## Supplementary Material


