# Supplementary material for: Safety outcomes of sodium-glucose cotransporter-2 inhibitors in patients with type 2 diabetes and other risk factors for cardiovascular disease: a systematic review and meta-analysis
Source: Cardiovasc Endocrinol Metab. 2023 May 8;12(2):e0284. doi: 10.1097/XCE.0000000000000284 (PMC10171571; doi:10.1097/XCE.0000000000000284)
Supplement: Supplementary file 1 [file xce-12-e0284-s001.pdf]

Supplementary Table S1: The modified Jaded Scale

Eight items	Answer	Score
Was the study described as randomized?	Yes	+1
	No	0
Was the method of randomization appropriate?	Yes	+1
	No	-1
	Not described	0
Was the study described as blinding?	Yes	+1
	No	0
Was the method of blinding appropriate?	Yes	+1
	No	-1
	Not described	0
Was there a description of withdrawals and dropouts?	Yes	+1
	No	0
Was there a clear description of the inclusion/exclusion criteria?	Yes	+1
	No	0

Supplementary Table S2 Modified Jadad Scores of the Included Studies

Study	Was the research describe d as randomi zati on?	Was the approach of randomiz ati on appropriat e ?	Was the researc h describ ed as blinding ?	Was the approach of blinding appropriat e?	Was there a presentati on of the withdraw a ls and dropouts?	Was there a presentati on the inclusion/ exclu sion criteria?	Was the approac h used to assess adverse effects describe d?	Was the approac h of statistica l analysis describe d?	Total
CANVAS Neal et al	1	1	1	1	1	1	1	1	8
DAPA-HF McMurray et al	1	1	1	1	1	1	1	1	8
DECLAR E-TIMI 58 Wiviott et al	1	1	1	1	1	1	1	1	8
EMPA-REG OUTCOM E Zinman et al	1	1	1	1	1	1	1	1	8
CREDEN CE Perkovic et al	1	1	1	1	1	1	1	1	8
DAPA-CKD, Heerspink et al	1	1	1	1	1	1	1	1	8

EMPEROR-Reduced, Packer et al	1	1	1	1	1	1	1	1	8
VERTIS-CV, Cannon et al	1	1	1	1	1	1	1	1	8
SOLOIST-WHF, Bhatt et al	1	1	1	1	1	1	1	1	8
SCORED, Bhatt et al	1	1	1	1	1	1	1	1	8

Supplementary Table S3: Definition of inclusion, exclusion, primary outcome, secondary outcome

Study Name	Inclusion Criteria	Exclusion criteria	Primary outcome	Secondary outcome
EMPAREG-Outcome (Type 2 Diabetes) Zinman et al	Type 2 diabetes<br><br>adults (≥18)<br><br>BMI of 45 or less<br><br>Glomerular filtration rate (GFR) >30<br><br>Established cardiovascular disease<br><br>Background glucose-lowering therapy unchanged for ≥12 weeks prior to randomization or, in the case of insulin, unchanged by >10% from the dose at randomization in the previous 12 weeks	Uncontrolled hyperglycemia with glucose >240 mg/dL after an overnight fast during placebo run- in and confirmed by a second measurement (not on the same day).<br><br>Indication of liver disease<br><br>Planned cardiac surgery or angioplasty within 3 months.<br><br>Estimated glomerular filtration rate <30 ml/min<br><br>Any uncontrolled endocrine disorder except type 2 diabetes	CV Death (Including Fatal Stroke and Fatal MI), Non-fatal MI (Excluding Silent MI), and Non-fatal Strok	composite of the primary outcome plus hospitalization for unstable angina.
CANVAS and CANVAS-R (Type 2 Diabetes), Neal et al	Type 2 diabetes (HgbA1c ≥7.0% and ≤10.5%)<br><br>greater than or equal to (>=) 30 yrs old with history of cardiovascular (CV) event, or >= 50 yrs old with high risk of CV events<br><br>Glomerular filtration rate (GFR) >30 ml/min	History of diabetic ketoacidosis, type 1 diabetes, pancreas or beta-cell transplantation, or diabetes secondary to pancreatitis or pancreatectomy.<br><br>H/o one or more severe hypoglycemic episode with in 6 months before screening.<br><br>MI or unstable angina, revascularization procedure, or cerebrovascular accident within 3 months before screening.<br><br>planned revascularization procedure<br><br>history of NYHA IV cardiac disease	composite of death from cardiovascular causes, nonfatal myocardial infarction, or nonfatal stroke.	death from any cause, death from cardio vascular causes, progression of albuminuria, composite of death from cardiovascular causes and hospitalization for heart failure

CREDENCE (type 2 DM and nephropathy) Perkovic et al	Age ≥30 years<br><br>type 2 diabetes, with HgbA1c of 6.5 to 12.0%<br><br>Estimated glomerular filtration rate (eGFR) ≥30 to <90 mL/min/1.73 m2 chronic kidney disease, defined as an eGFR (of 30 to <90 ml/min)<br><br>Urinary albumin: creatinine ratio (UACR) >300 mg/g to ≤5000 mg/g (>33.9 mg/mmol to ≤565.6 mg/mmol)<br>stable maximum tolerated labelled daily dose of ACEi or ARB for at least 4 weeks prior to randomization	History of diabetic ketoacidosis or type 1 diabetes mellitus (T1DM)<br><br>History of hereditary glucose-galactose malabsorption or primary renal glucosuria<br><br>Known medical history or clinical evidence suggesting nondiabetic renal disease<br><br>Renal disease that required treatment with immunosuppressive therapy or a history of chronic dialysis or renal transplant<br><br>Uncontrolled hypertension (systolic blood pressure [BP] ≥180 and/or diastolic BP ≥100 mmHg)<br>Myocardial infarction, unstable angina, revascularization procedure (e.g.,stent or bypass graft surgery), or cerebrovascular accident within 12 weeks before randomization, or a revascularization procedure is planned during the trial	composite of end-stage kidney disease, doubling of the serum creatinine level from baseline or death from renal or cardiovascular disease.	composite of cardiovascular death or hospitalization for heart failure, composite of cardiovascular death, myocardial infarction, or stroke, hospitalization for heart failure, cardiovascular death, death from any cause, composite of end-stage kidney disease, doubling of the serum creatinine level, or renal death, composite of cardiovascular death, myocardial infarction, stroke, or hospitalization for heart failure or for unstable angina

		Current or history of heart failure of New York Heart Association (NYHA) class IV cardiac disease		
DAPA-HF (HFrEF) McMurray et al	Age ≥18 years<br><br>Ejection fraction of ≤40%<br><br>New York Heart Association Class II-IV symptoms<br><br>Plasma NT-proBNP level of:<br>≥ 600pg/mL OR<br>≥ 400pg/mL if they were hospitalized for HF within the past 12 months OR<br><br>≥ 900pg/mL if patient had atrial fibrillation/flutter on baseline ECG	Receiving therapy with an SGLT2 inhibitor within 8 weeks prior to enrolment or previous intolerance of an SGLT2 inhibitor<br><br>Type 1 diabetes mellitus<br><br>Symptoms of hypotension or SBP < 95 mm Hg<br><br>estimated glomerular filtration rate <30 ml/min/1.73 m2	composite of worsening heart failure or death from cardiovascular causes.	composite of hospitalization for heart failure or cardiovascular death, total number of hospitalizations for heart failure (including repeat admissions) and cardiovascular deaths; the change from baseline to 8 months in the total symptom score on the Kansas City Cardiomyopathy Questionnaire
DECLARE - TIMI 58 (Type 2 diabetes) Wiviott et al	Age ≥40 years<br><br>type 2 diabetes, HgbA1c of at least 6.5% but less than 12.0%,<br><br>creatinine clearance of 60 ml or more per minute.<br><br>multiple risk factors for atherosclerotic cardiovascular disease or established atherosclerotic cardiovascular disease<br><br>Or<br><br>No known cardiovascular disease AND at least two cardiovascular risk factors in addition to T2DM	Diagnosis of type 1 DM<br>History of bladder cancer or history of radiation therapy to the lower abdomen or pelvis at any time<br><br>Chronic cystitis and/or recurrent urinary tract infections<br><br>Pregnant or breast-feeding patients	Safery outcome: MACE(defined as cardiovascular death, myocardial infarction, or ischemic stroke) efficacy outcome: MACE and a composite of cardiovascular death or hospitalization for heart failure.	renal composite outcome, defined as a sustained decrease of 40% or more in estimated glomerular filtration rate (eGFR), death from any cause

DAPA-CKD (CKD+- DM) Main outcome renal,2 outcomes interest, Heerspink et al	Age ≥18 years old<br>With or without type 2 diabetes<br><br>eGFR ≥25 and ≤75 mL/min/1.73 m2<br><br>urinary albumin-to-creatinine ratio 200 to 5000 mg/g on visit 1<br><br>Stable, and for the patient maximum tolerated labelled daily dose, treatment with ACE-I or ARB for at least 4 weeks before visit 1, if not medically contraindicated	polycystic kidney disease, lupus nephritis,ANCA-associated vasculitis<br><br>Receiving immunotherapy for primary or secondary renal disease within 6 months<br><br>History of organ transplantation<br><br>Use of SGLT2 inhibitor within 8 weeks prior or previous intolerance of an SGLT2 inhibitor<br><br>Type 1 diabetes mellitus<br><br>New York Heart Association (NYHA) class IV Congestive Heart Failure<br><br>MI, unstable angina, stroke or transient ischemic attack within 12 weeks prior to enrolment	composite of a sustained decline in the estimated GFR of at least 50%, end-stage kidney disease, or death from renal or cardiovascular causes.	composite kidney outcome of a sustained decline in the estimated GFR of at least 50%, end stage kidney disease, or death from renal causes; a composite cardiovascular outcome defined as hospitalization for heart failure or death from cardiovascular causes; and death from any cause.
EMPEROR-Reduced, Packer et al	≥18 years of age<br><br>chronic heart failure (functional class II, III, or IV) with LVEF of 40% or less<br><br>receiving appropriate treatments for heart failure, including diuretics, inhibitors of RAS and neprilysin, beta-blockers, mineralocorticoid receptor antagonists, and, when indicated, cardiac devices.	-Myocardial infarction, coronary artery bypass graft surgery, or other major cardiovascular surgery, stroke or TIA (Transient Ischemic Attack) in past 90 days prior to Visit 1<br>-Heart transplant recipient, or listed for heart transplant<br>-Acute decompensated HF<br>-Systolic blood pressure (SBP) >= 180 mmHg at Visit 2.<br>-Symptomatic hypotension and/or a SBP < 100 mmHg<br>-Indication of liver disease<br>-Impaired renal function, defined as eGFR (Estimated Glomerular Filtration Rate) < 20 mL/min/1.73 m2 (CKD-EPI (Chronic Kidney Disease - Epidemiology Collaboration Equation)) or requiring dialysis<br>-History of ketoacidosis<br>-Current use or prior use of a SGLT (Sodium-glucose co-transporter)-2 inhibitor or	composite of cardiovascular death or hospitalization for worsening heart failure.	occurrence of all adjudicated hospitalizations for heart failure, including first and re-current events. , rate of the decline in the estimated GFR during double-blind treatment.

		combined SGLT-1 and 2 inhibitor<br>-Currently enrolled in another investigational device or drug study<br>-Known allergy or hypersensitivity to empagliflozin or other SGLT-2 inhibitors<br>-Women who are pregnant, nursing, or who plan to become pregnant while in the trial		
VERTIS-CV, Cannon et al	Age ≥40 years<br><br>type 2 diabetes (with HgbA1c of 7.0 to 10.5%)<br><br>established atherosclerotic cardiovascular disease involving the coronary, cerebrovascular, or peripheral arterial systems.<br><br>Stable on allowable antihyperglycemic agents (AHAs) or on no background AHA for ≥8 weeks prior to study participation	history of type 1 diabetes or ketoacidosis<br><br>estimated glomerular filtration rate below 30 ml per minute per 1.73 m2 of body-surface area.<br><br>Experiencing a cardiovascular event (myocardial infarction or stroke) or undergoing coronary angioplasty or peripheral intervention procedure<br><br>Undergoing any cardiovascular surgery (valvular surgery) within 3 months of study participation<br><br>Planned revascularization or peripheral intervention procedure or other cardiovascular surgery<br><br>New York Heart Association (NYHA) IV heart failure at study participation<br>.	Death from cardiovascular causes, nonfatal myocardial infarction, or nonfatal stroke	Tcomposite of death from cardiovascular causes or hospitalization for heart failure; death from cardiovascular causes, composite of death from renal causes, renal replacement therapy, or doubling of the serum creatinine level.

SOLOIST-WHF, Bhatt et al	Age 18 to 85 years<br><br>hospitalized because of the presence of signs and symptoms of heart failure and received treatment with intravenous diuretic therapy and diagnosis of type 2 diabetes before the index admission or to have laboratory evidence to support a diagnosis of type 2 diabetes during the index admission.	end-stage heart failure or recent acute coronary syndrome, stroke, percutaneous coronary intervention or coronary artery bypass surgery, or an estimated GFR of less than 30 ml per minute per 1.73 m2 of body surface area.	Deaths from cardiovascular causes and hospitalizations and urgent visits for heart failure)	The revised secondary endpoints were the total number of hospitalizations and urgent vis-<br>its for heart failure; the incidence of death from cardiovascular causes; the incidence of death from any cause; the total number of deaths from cardiovascular causes, hospitalizations for heart failure, nonfatal myocardial infarctions, and nonfatal strokes; the total number of deaths from cardiovascular causes, hospitalizations and urgent visits for heart failure, and events of heart failure during hospitalization; the change in score on the Kansas City Cardiomyopathy Questionnaire–12 item (KCCQ-12; scores range from 0 to 100, with higher scores indicating better quality of life) to month 4; and the change in the estimated GFR.31
SCORED, Bhatt et al	Persons 18 years of age or older with type 2 diabetes mellitus with a glycated hemoglobin level of 7% or	-Antihyperglycemic treatment has not been stable within 12 weeks prior to screening.	The primary endpoint was changed during the trial to the	-Total no. or hospitalizations for HF and urgent visits for HF

	higher, chronic kidney disease (eGFR, 25 to 60 ml per minute per 1.73 m2 of body-surface area), and additional cardiovascular risk factors were enrolled. The risk factors consisted of at least one major cardiovascular risk factor in those18 years of age or older or at least two minor cardiovascular risk factors in those 55 years of age or older. An exclusion criterion was any plan to start an SGLT2 inhibitor during the trial.	-Planned coronary procedure or surgery after randomization.<br>-Lower extremity complications (such as skin ulcer, infection, osteomyelitis, and gangrene) identified during screening and requiring treatment at randomization.<br>-Planning to start a sodium-glucose linked transporter-2 (SGLT2) inhibitor during the study.	composite of the total number of deaths from cardiovascular causes, hospitalizations for heart failure, and urgent visits for heart failure.	Deaths from cardiovascular causes Total no. of deaths from cardiovascular causes, hospitalizations for HF, nonfatal myocardial infarctions, and nonfatal strokes<br>-Total no. of deaths from cardiovascular causes, hospitalizations for HF, urgent visits for HF, and events of HF during hospitalization<br>-First occurrence of a sustained decrease of ≥50% in the eGFR from baseline for ≥30 days, long-term dialysis, renal transplantation, or sustained eGFR of <15 ml/min/1.73 m2 for ≥30 days<br>-Deaths from any cause Total no. of deaths from cardiovascular causes, nonfatal myocardial infarctions, and nonfatal strokes

Supplement Figure S4

Visual evaluation of the funnel plot shows no evidence of publication bias.

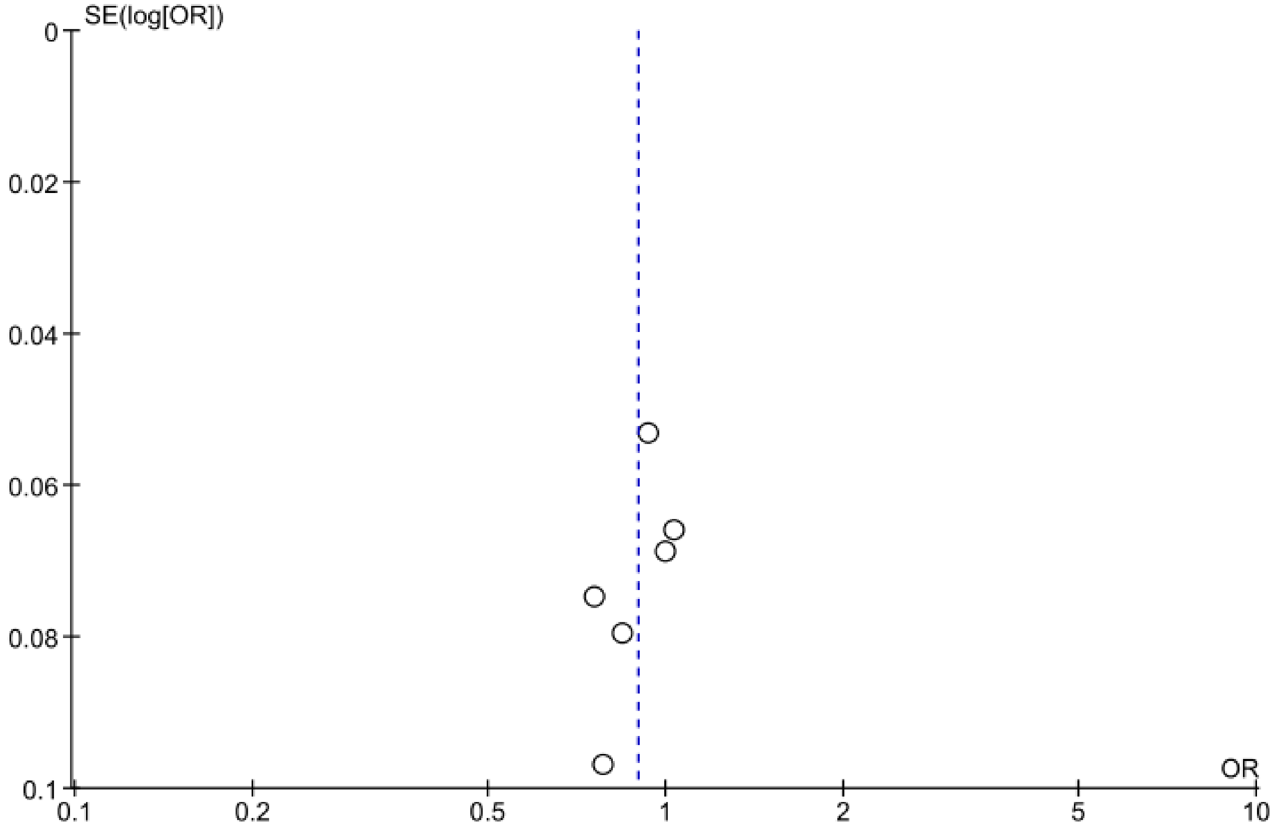

SE(log[OR])
0
0.02
0.04
0.06
0.08
0.1
0.1
0.2
0.5
1
2
5
10
OR
